# PANCREATECTOMY AND HEPATECTOMY: A COMBINED SURGICAL RESECTION OF PSEUDOPAPILLARY SOLID TUMOR OF PANCREAS ASSOCIATED WITH HEPATIC METASTASIS

**DOI:** 10.1590/0102-672020220002e1703

**Published:** 2022-12-19

**Authors:** Enio Campos Amico, Gustavo Rêgo Coelho, José Sandro Pereira da Silva, Clara Wilma Fernandes Rosendo, Mariana Bezerra Teles, José Huygens Parente Garcia

**Affiliations:** 1 University Hospital Onofre Lopes – Natal (RN), Brazil;; 2 University Hospital Walter Cantídio – Fortaleza (CE), Brazil.

**Keywords:** Pancreatic Neoplasms, Neoplasm Metastasis, Liver Neoplasms, Abdominal Neoplasms, Neoplasias pancreáticas, Metástase Neoplásica, Neoplasias Hepáticas, Neoplasias Abdominais

## Abstract

**BACKGROUND:**

Solid pseudopapillary tumor of the pancreas has been frequently reported in the past two decades. Surgery remains the treatment of choice, with the liver being the most frequent site of metastases.

**AIMS:**

The study aimed to present an option of surgical treatment for an 18-year-old female patient with a solid lesion in the body and tail of the pancreas associated with metastatic lesions in both hepatic lobes.

**METHODS:**

Two surgical procedures were scheduled. In the first procedure, body-caudal pancreatectomy with splenectomy was performed, associated with the resection of three lesions of the liver's left lobe. A right hepatectomy was performed 6 months later, progressing without complications.

**RESULTS:**

The patient continues without clinical complaints on the last return, and abdominal magnetic resonance performed 28 months after the second procedure does not show liver or abdominal cavity lesions.

**CONCLUSIONS:**

The knowledge on the biological behavior of tumors, evolution, and recurrence risks allows the indication of more rational surgical techniques that best benefit patients.

## RESUMO

**RACIONAL:**

O tumor sólido pseudopapilar do pâncreas tem sido relatado na literatura com mais frequência nas últimas duas décadas. A cirurgia continua sendo o tratamento indicado, sendo o fígado o local mais frequente de metástases.

**OBJETIVOS:**

Apresentar a opção de tratamento cirúrgico de paciente feminino, 18 anos, com lesão sólida no corpo e cauda do pâncreas associada a lesões metastáticas em ambos os lobos hepáticos.

**MÉTODOS:**

Dois procedimentos cirúrgicos foram agendados. No primeiro procedimento foi realizada pancreatectomia corpo-caudal com esplenectomia, associada à ressecção de 3 lesões do lobo esquerdo do fígado. A hepatectomia direita foi realizada seis meses após, evoluindo sem complicações.

**RESULTADOS:**

O paciente continua sem queixas clínicas no último retorno, e a ressonância magnética do abdomem realizada 28 meses após o segundo procedimento não mostra lesões hepáticas ou na cavidade abdominal.

**CONCLUSÕES:**

O conhecimento do comportamento biológico dos tumores, a evolução e os riscos de recidiva, permitem a indicação de técnicas cirúrgicas mais racionais e que melhor beneficiam os pacientes.

## INTRODUCTION

First described by Frantz in 1959, pseudopapillary solid tumor (SPT) of the pancreas has been more frequently reported in the literature in the past two decades^2^. For this type of tumor, surgery remains the indicated treatment, and the prognosis is considered excellent^2^. Some authors, however, have described that 10–15% presented malignant behavior, and its presentation can be aggressive with the invasion of adjacent structures or even distant metastases^3^. Although the liver is the most frequent site of metastases, there is no evidence on the best type of treatment in this scenario.

The objective of this study was to describe the technical option indicated in a young patient with an SPT in the body and tail of the pancreas and both lobes of liver metastases and to discuss the benefit of this type of approach based on a literature review.

## METHODS

A female patient, aged 18 years, with epigastric pain for 6 months, with no findings on physical examination, and with a negative medical history for other diseases, was admitted for routine examination. The general laboratory tests showed no changes (Ca 19-9: 1.6 U/mL; carcinoembryonic antigen: 1.1 ng/mL). Abdominal tomography revealed a solid lesion on the body and tail of the pancreas, oval, with lobulated contours and heterogeneous post-contrast enhancement measuring 6.2×4.4, with small coarse calcifications inside. In the liver, two solid confluent lesions were identified with heterogeneous enhancement by hypodense areas compatible with necrosis inside them in the right hepatic lobe [segments VII (11.2×7.7 cm) and V/VI (8.3×8.3 cm)]. Another three lesions in the left lobe of the liver were also found: one in segment III (7.6×5.0 cm) and other two in segment II (6.0×5.0 and 1.5×1.0 cm). There were at least two enlarged and necrotic lymph nodes between the stomach and the spleen. A chest CT scan showed no abnormalities.

**Figure 1:**
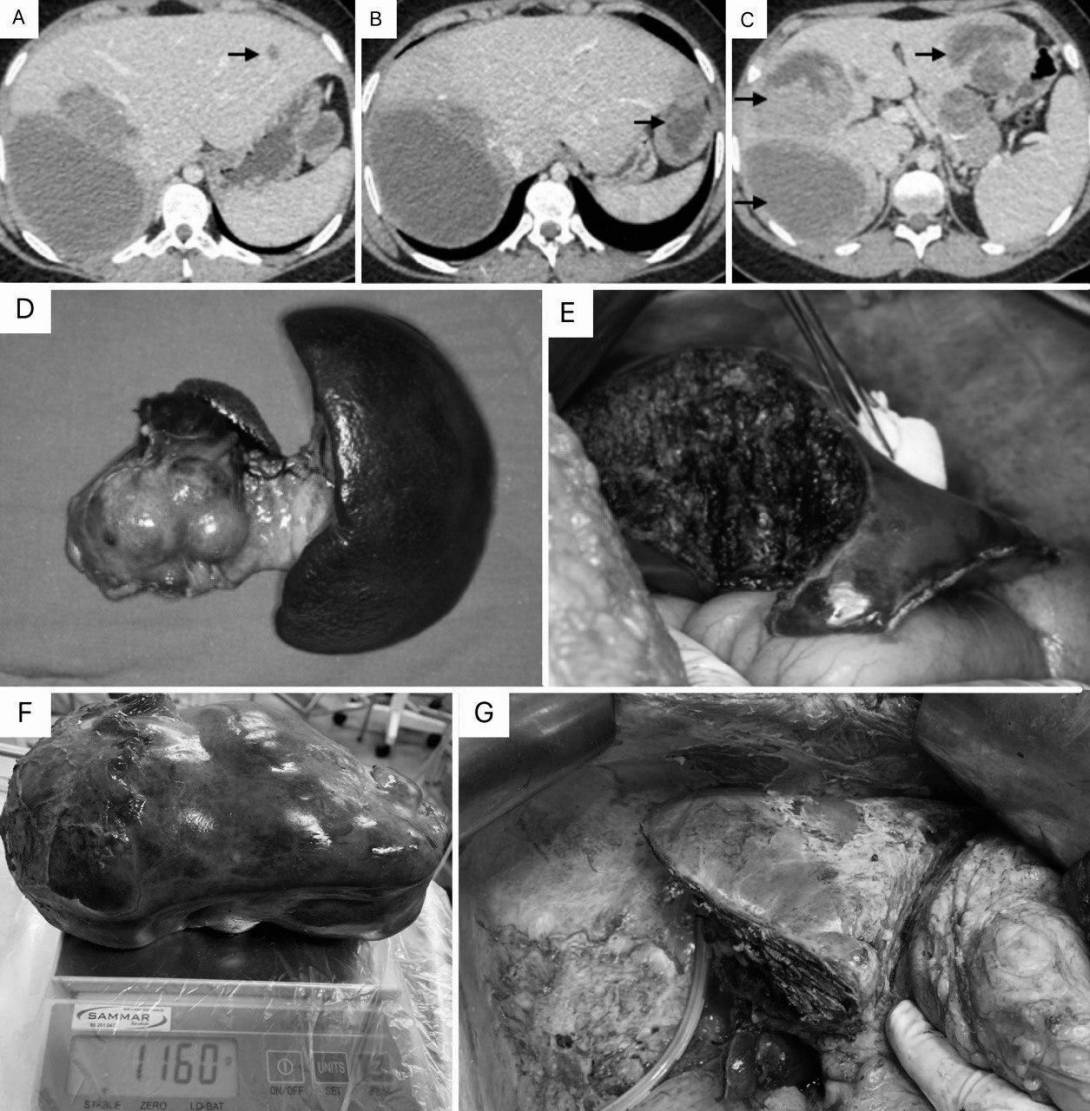
(A–C) Portal phase of abdominal tomography showing hypodense lesions on the black arrows. It is possible in larger lesions to identify a solid-cystic component of the lesions; (D) Body-caudal pancreatectomy and splenectomy (first surgery); (E) Intraoperative image of the left hepatic lobe after resection (first surgery); (F) Surgical specimen after right hepatectomy (second surgery) (weight: 1160 g); (G) Final appearance of the remaining liver (second surgery).

The patient underwent a percutaneous liver biopsy whose immunohistochemical examination revealed metastasis of low-grade epithelioid neoplasia compatible with SPT. The surgical procedures were carried out in two stages, and the patient received preoperative vaccination to prevent sepsis after splenectomy. In the first procedure (December 2018, Onofre Lopes University Hospital), after an intraoperative ultrasound, a body-caudal pancreatectomy was performed with splenectomy associated with the resection of three lesions of the half-left liver (size: 9×6, 6×5, and 1.5×1.0 cm) (Figure 1). The patient developed an intraperitoneal abscess that was treated by antibiotic therapy and percutaneous drainage. The histopathological analysis confirmed an SPT (5×4 cm) with liver metastases, negative lymph nodes (10/10), and disease-free margins. A new abdominal magnetic resonance imaging (MRI) showed that the lesions in the right hepatic lobe remained stable, and the second surgical procedure was scheduled. The patient underwent a right hepatectomy 6 months after the first procedure (June 2019, Walter Cantídeo University Hospital), without complications. Informed consent of the patient was approved for this publication.

## RESULTS

The patient continues without clinical complaints on the last return, and an abdominal MRI performed 28 months after the second procedure does not show liver or abdominal cavity lesions.

## DISCUSSION

Since, in most patients with SPT, the disease is localized and the resection is efficient, it is natural to think of a role for combined resection surgical procedures in the setting of primary disease associated with metastases. Imaging tests are very important for differential diagnosis with other tumors^1^. However, due to the rarity of the aggressive form of SPT, only a few case reports and isolated institutional experiences on management in this scenario have been published. These records are one more that corroborate the validity of these kinds of procedures.

In 2002, Law et al. recorded the successful treatment of liver metastases resections in four patients with SPT^4^ who were treated at the Memorial Sloan-Kettering Cancer Center. Two patients were alive for more than 5 years after the surgery, and the other had no recurrence 11 years after the procedure.

In 2014, a study in the Pecking Union Medical College Hospital also evaluated four patients with SPT with hepatic metastasis in the post-pancreatectomy follow-up (three cases) or associated with pancreatectomy (one case) and who underwent hepatectomy^5^. It is noteworthy that, except for patients with synchronic disease, in all the others, there was local recurrence (pancreas, retroperitoneum, and lymph nodes) associated with metastases that were also removed during the surgical procedure. The size and number of metastases varied between 1.5 and 8.1 cm and between 1 and 7 lesions, respectively. There was recurrence with a segment at 1, 3, 23, and 64 months after surgical resections in none of the cases. Despite its occurrence in two patients, the liver metastasis resection in this study was promising even in a scenario of extrahepatic disease.

In a recent study, which corresponds to the largest Western casuistic of patients with metastatic SPT, 24 out of 340 patients were analyzed from the National Cancer Database from 1998 to 2011^6^. The main sites of metastasis in patients were the liver and lung, and, in 7 (29%) of the 24 cases, patients underwent resection. Although there was worse survival in metastatic cases when compared with patients without metastases, surgical resection was associated with extended survival even in this group. Most importantly, among the seven patients with metastases who underwent surgery, survival was similar to that of other nonmetastatic patients.

Hao et al., in 2018, carried out a literature review that included 59 patients with aggressive SPT collected from case reports^2^. The main reason for considering the cases as “aggressive” was a systemic metastatic disease present in 81.4% of the cases. Of the total number of patients included, liver metastases were identified in 39 (66.1%) patients. The rate of curative resections in the whole series was 91.5%. The study pointed to an average survival of 152.67±12.8 months with a survival rate of 71.7% in 5 years and 65.5% in 10 years. Such survival rates were obtained despite 69.5% of the cases presenting recurrence or new metastases in the follow-up, which, according to the authors, conferred a favorable course of the disease with a treatment aimed at the resection of the metastatic disease.

Most of the studies are heterogeneous, retrospective, including a small number of patients, and sometimes based on isolated case reports, which implies an expected bias in selecting favorable cases. Nonetheless, together they suggest that in some of the cases in which SPT is associated with liver metastases, the combined surgical resection can cure or, when not, at least a more prolonged survival and, therefore, a better prognosis for patients undergoing the procedure. In this sense, the present report, although with a short follow-up period (15 months), seems to corroborate the literature findings.

When offering the alternative of resection surgery for cancer in the context of systemic disease, it is natural that the patient's cancer prognosis be evaluated. This is common, for example, in colorectal liver metastases or in liver metastases in patients with neuroendocrine tumors. It is important to know the tumor's behavior and try to avoid procedures in those cases where the benefit of the surgery will be incompatible with the risks. From this perspective, studies exploring the risk factors for recurrence of SPT have been published over the years with conflicting results^8^. More recently, however, based on the description of biological similarity between SPT and neuroendocrine tumors^7^, studies were able to establish a simple and easy-to-apply prognostic index. Studying a cohort of 193 patients with operated SPT, the same authors observed in the multivariate analysis that the tumor size and the Ki-67 index were independent predictive factors for recurrence-free survival. Based on the combination of these factors, they were more accurately defined than the American Joint Committee on Cancer and European Neuroendocrine Tumor Society TNM staging systems, an efficient classification for disease recurrence. The authors recommend that, due to the rarity of the SPT, multi-institutional studies should be carried out to validate this new proposed system^7^.

## CONCLUSION

The resection of liver metastases in patients with SPT seems to be justified by a conjunction of two favorable factors: the first is the well-known indolent course of SPT even in metastatic disease, and the second is the fact that SPT occurs in young patients who can tolerate major surgical procedures with curative intent. The experience with combined resections of the primary tumor and liver metastases has effectively cured some of the patients while promoting a better survival time in those in which relapse occurs. Associated with this, the better knowledge on the biological behavior of tumor and its risks of recurrence from the recent application of more efficient prognostic indexes promises a modern and more rational approach to the surgical indication of these patients.
